# Intermittent Visual Occlusions Increase Balance Training Effectiveness

**DOI:** 10.3389/fnhum.2022.748930

**Published:** 2022-04-25

**Authors:** Evangelia-Regkina Symeonidou, Daniel P. Ferris

**Affiliations:** ^1^J. Crayton Pruitt Family Department of Biomedical Engineering, University of Florida, Gainesville, FL, United States; ^2^International Max Planck Research School for Systems and Cognitive Neuroscience, University of Tübingen, Tübingen, Germany

**Keywords:** gait, visuomotor, motor learning, adaptation, balance beam, stroboscopic glasses

## Abstract

Improving dynamic balance can prevent falls in humans with neurological and mechanical deficits. Dynamic balance requires the neural integration of multisensory information to constantly assess the state of body mechanics. Prior research found that intermittent visual rotations improved balance training during walking on a narrow beam, but limitations from the immersive virtual reality headset hindered balance training effectiveness overall. We theorized that intermittent visual occlusions with electrically controlled liquid crystal glasses would overcome the previous limitations of the immersive virtual reality headset and provide a means to enhance dynamic balance training efficacy. Forty healthy young individuals walked on a treadmill-mounted balance beam for 30 min (20 subjects with intermittent visual occlusions and 20 subjects with unperturbed vision). Balance performance, in number of step-offs of the beam, improved by 78% for the visual occlusions group on the same day of the training, a near fourfold improvement compared to the 21% improvement for the unperturbed vision group (*t*(38) = –5.2, *p* < 0.001). The difference between groups was also apparent 2 weeks later testing for retention (60% improvement for the visual occlusions group, 5% for the unperturbed vision group; *t*(38) = –4.2, *p* < 0.001). Intermittent visual occlusions are likely a simple method for enhancing balance training in dynamic motor tasks.

## Introduction

Falls are a major public health concern with socioeconomic consequences including high medical bills and fatalities (Masud and Morris, 2001). Most falls occur during locomotion, especially when individuals fail to predict or react to changes in their environment (Berg et al., 1997; Talbot et al., 2005). A survey on seniors’ falls in Canada reported that in 2001/02 “Fifty- nine percent (*N* = 67,876) of the fall-related hospital admissions were for people age 65 years and over” (Scott et al., 2005). As the average population age continues to rise there is a need to develop appropriate training interventions to improve dynamic balance control and reduce the risk of falls.

Beam walking proficiency can reflect walking balance ability across different populations (Sawers and Ting, 2015). It is a skill requiring considerable dynamic balance control by integrating information of different sensory modalities. It is also a suitable clinical test for assessing balance during gait and predicting falls in older individuals and individuals with neurological impairments, since it requires the center of mass to remain within the base of support similar to walking (Hortobágyi et al., 2019). A recent study from our lab showed that training individuals with brief, intermittent visual perturbations improved balance beam performance (Peterson et al., 2018). Healthy, young participants were presented with brief reoccurring visual rotations in the roll axis, delivered through a virtual reality headset. The group training with the visual rotations had an immediate reduction in step-offs of 42%, compared to the group without visual rotations’ reduction of 9%. However, neither of the virtual reality groups improved as much as the group training without any virtual reality headset (44%). This is likely due to a combination of the low visual refresh rate, the reduced peripheral vision, and/or the delay between visual and vestibular feedback caused by the virtual reality headset. Introduction of visual perturbations without the limitations from the immersive virtual reality headset might be able to enhance balance training beyond that demonstrated in the previous study (Peterson et al., 2018).

One method that athletes use for introducing visual perturbations is training with stroboscopic liquid crystal glasses. Professional field hockey players that underwent one session of stroboscopic training showed an 18% performance increase compared to participants that trained under normal visual conditions (Mitroff et al., 2013). Similarly, professional badminton players that underwent a 4-week stroboscopic training protocol, showed a ∼10% higher proportion of successful hits compared to the control group after (Hülsdünker et al., 2019). Participants showed an 18% higher increase in ball catching performance after 8 sessions of stroboscopic training compared to the control group (Holliday, 2013). Stroboscopic vision may also have benefits in promoting sensory re-weighting during balance tests and improving postural control with training (Kim et al., 2017; Lee et al., 2021). The studies above indicate that visual occlusions provide a benefit for a variety of motor tasks and could be useful for training dynamic balance.

The goal of our study was to determine if brief, intermittent visual occlusions could be used as a means to enhance balance training. To overcome the previously discovered limitations from using an immersive virtual reality headset, we studied healthy young subjects walking on a treadmill mounted balance beam while wearing electrically controlled liquid crystal glasses. In one group of subjects, the glasses provided transient, intermittent visual occlusions lasting 1.5 s, followed by 7.5 s of clear vision. A second group of subjects wore the glasses but had no occlusions during training. We hypothesized that the group with intermittent visual occlusions would have greater reductions in the number of step offs when comparing a pre-training evaluation with a post-training evaluation. Subjects walked on the balance beam for one 30-min session in between pre-and post-training evaluation. We also tested the subjects again 2 weeks later (retention) to determine if the training effects were still evident in balance performance.

## Materials and Methods

We recruited forty right leg dominant, healthy, young participants (20 males, 20 females). Leg dominance was assessed by asking individuals which foot they would use to kick a ball. Participants reported having no neurological, orthopedic, or musculoskeletal condition or lower limb surgeries. The experiment was approved by the institutional review board of the University of Florida and all participants had to read and sign the informed consent form prior to their participation in the experiment.

**FIGURE 1:**
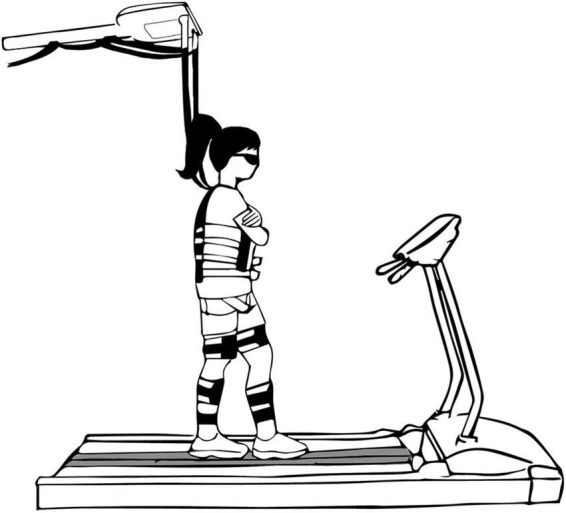
A sketch of a participant walking on the treadmill-mounted balance beam while wearing the occlusion glasses and safety harness. The participants wore a harness for safety that allowed for freedom of movement in the mediolateral direction.

All participants had to train their balance while tandem walking on a treadmill-mounted beam that was 2.5 cm high and 2.5 cm wide (Domingo and Ferris, 2009; Sipp et al., 2013; Peterson and Ferris, 2018; Peterson et al., 2018) while wearing occlusion glasses (Senaptec Strobe, Senaptec, Oregon, United States), which had the ability to change from clear to opaque, restricting their vision (Figure 1). The treadmill was set to move at a fixed speed of 0.22 m/s, which was based on a previous study in our lab (Domingo and Ferris, 2009; Peterson and Ferris, 2018; Peterson et al., 2018). Participants were randomly assigned to one of two training protocols: (a) training with visual occlusions (10 females, 10 males, age = 25.1 ± 4.7), (b) training with unperturbed vision (10 females, 10 males, age = 24.7 ± 4.5). The visual occlusions group was presented with periodic visual occlusions of 1.5 s followed by 7.5 s of clear vision, while the unperturbed vision group performed the training without any visual occlusions. The training lasted a total of 30 mins and consisted of three 10-min trials. Both groups also performed a 3-min pre-test before the training and 3-min post-test on the same day and a 3-min retention trial 2 weeks later. The pre-test, post-test, and retention trial were performed with the glasses on and without any occlusions, for both groups. Participants were able to take breaks between training trials as needed and had to take a 5-min break between the last training trial and the post-test trial. They were strapped to a body harness for safety that was modified to increase freedom of movement in the mediolateral direction. The body harness was attached to a body-weight support system (Figure 1). Also, one experimenter was next to the treadmill at all times to assist the participants or start and stop the treadmill if needed.

To present the occlusions we connected the occlusion glasses *via* Bluetooth to a Samsung Note 10 (Samsung, Seoul, South Korea) that was running the Senaptec app (Senaptec, Oregon, United States). We then used TeamViewer to access the screen of the phone over a desktop computer. With a custom MATLAB script, we initiated a periodic cycle of a 1.5 s long occlusion followed by 7.5 s of clear vision.

Every time the participants stepped with both feet on the beam, we initiated the cycle with a button press and disrupted it when the participant stepped off the beam. When the participant was off the beam, normal vision was restored, until they stepped back on, and the procedure was repeated. To avoid any cognitive anticipatory effects, we used up to a 1 s delay for the occlusion onset. While beam walking, participants were instructed to cross their arms and look straight ahead at a fixation point. They were instructed to only move their hips side to side to balance, avoiding any body rotations across their longitudinal axis. Every time participants stepped off the beam, they were instructed to take 5 steps on the treadmill before stepping back on. The above instructions were implemented in previous studies of our lab and were a result of rigorous piloting (Domingo and Ferris, 2009; Peterson and Ferris, 2018; Peterson et al., 2018). It allowed the participants to steady themselves before stepping back on the beam and provided the experimenter enough time to re-initiate the occlusion cycle.

We evaluated performance by counting the step-offs per minute (Domingo and Ferris, 2009; Peterson and Ferris, 2018; Peterson et al., 2018). Specifically, we recorded the times the participant stepped off the beam divided by the time they spent on the beam. Step-offs were manually logged by the experimenter through a button press, any time the participant made contact with the treadmill. To assess balance improvement percentage for each group, we calculated the step-offs/min difference between the pre-test and post-test trials (same day), and between the pre-test and retention trial. To normalize across different subject skill levels, we divided both values by the pre-test step-offs/min baseline.

To determine if there was a main effect of the visual occlusions training on balance improvement percentage, we performed a repeated measures ANCOVA with the training (visual occlusions, unperturbed vision) as the between-subjects variable. Time of testing (same day, retention) was set as the within-subjects variable to evaluate long-term learning effects. To account for performance differences between the groups before training, we set the pre training step-offs/min as a covariate. We also used independent *t*-tests between the two groups for each day to evaluate performance differences between groups for both time points. A subset of the subjects wore EEG electrodes during testing for future analysis. *Post hoc* analysis of behavioral results showed there was no significant effect of the EEG equipment on the results.

## Results

**FIGURE 2:**
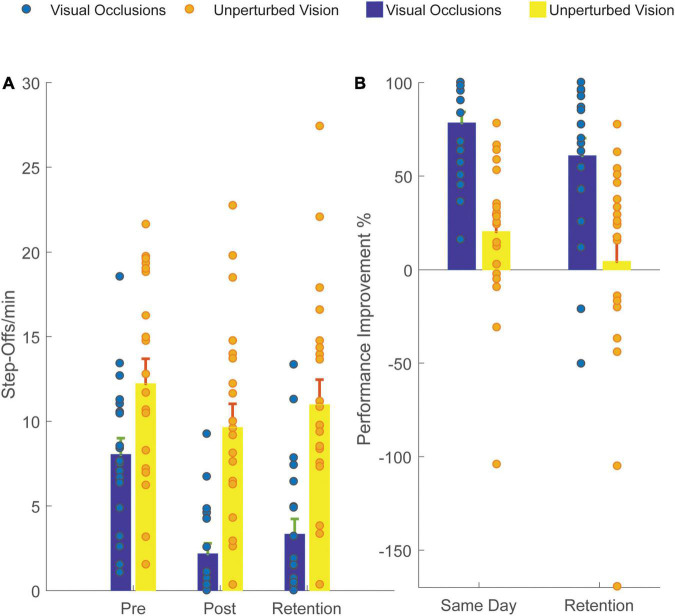
Balance Improvement for the Unperturbed Vision group (light colors – yellow/orange) and the Visual Occlusions group (dark colors – blue/green). **(A)** Step-Offs/min on the Same Day before (Pre) and after (Post) the Training and Two Weeks Later. **(B)** Performance improvement was calculated from the difference between Pre Step-Offs/min and Post Step-Offs/min, normalized to Pre Step-offs/min value. The intermittent visual occlusions improved training outcomes compared to unperturbed vision (ANOVA, *p* < 0.001). The error bars represent the standard error of the mean. Visual Occlusions had a fourfold percentage performance improvement compared to not having visual perturbations.

Training with intermittent visual occlusions improved dynamic balance (as indicated by a reduction in step-offs) more than training with unperturbed vision – there was a significant main effect of intervention (*F*_1, 38_ = 18.9, *p* < 0.001). The group with intermittent visual occlusions substantially reduced their step-offs (post) and had an improvement in balance by 78% (Standard Deviation ± 26%). Forty percent of the visual occlusions group experienced zero step-offs after training. In comparison, the unperturbed vision group showed a slight reduction in step-offs and only improved by ∼20% immediately after the training (Standard Deviation 41%; *t*_38_ = –5.2, *p* < 0.001; Figure 2). The difference was also apparent during retention testing (*t*_38_ = –4.2, *p* < 0.001). There was a significant main effect of testing time (*F*_1,18_ = 6.9, *p* < 0.05). Both groups showed less improvement over the initial baseline after 2 weeks compared to improvements on the same day, but the intermittent visual occlusions improved retention of the dynamic balance training compared to unperturbed vision. The visual occlusions group showed a 61% (Standard Deviation ± 42%) performance improvement retention while the unperturbed vision group showed a 5% (Standard Deviation ± 59%) performance improvement retention. There was no significant interaction between testing time and training intervention. The within-subject effect size for the pre- to post-test difference within the group with intermittent visual occlusions was 1.2, while the between-subject effect size for the comparison of outcomes between the two groups was 1.5 (Lesinski et al., 2015).

## Discussion

The enhanced motor performance by the visual occlusions group, both on the training day and 2 weeks later (retention), suggests intermittent visual occlusions can be an effective means of training human balance at least in younger adults. Compared to the unperturbed vision group, the visual occlusions group showed a twofold reduction in step-offs/min on the same day (unperturbed vision: pre = 12.2 and post = 9.6; visual occlusions: pre = 8 and post = 2.1), which translated in a fourfold relative performance improvement when comparing the two groups (unperturbed vision: same day = 21%; visual occlusions: same day = 78%; Table 1). When testing for retention, the difference between groups was even bigger, with the unperturbed vision group experiencing 11 step-offs/min (pre = 12.2) while the visual occlusions group experienced only 3.3 step-offs/min (pre = 8.04). The visual occlusions group retained most of the performance improvement they had gained on the same day (78% improvement on the first day and 61% on the two-week follow-up; Figure 2). In contrast, the unperturbed vision group only retained 23% of the performance improvement that they had gained on the same day (21% improvement on the first day and 5% on the two-week follow-up). These values suggest that the intermittent visual occlusions not only improved the acquisition of a balance skill but also improved retention of the new balance ability.

**TABLE 1:** Step–Offs/Min and Percentage Improvement for the Unperturbed Vision and Visual Occlusions Group.

		Step-Offs/min			% performance improvement	
		Pre	Post	Retention	Same day	Retention
Unperturbed vision	Mean	12.22	9.63	10.98	20.47	4.54
	STD	6.57	6.25	6.64	40.58	59.33
Visual occlusions	Mean	8.04	2.17	3.34	78.43	60.89
	STD	4.31	2.77	4.02	26.28	41.94

*Mean and standard deviation (STD) of step-offs/min for pre and post training on the same day and for retention testing for each group (left). Mean and standard deviation of percentage performance improvement after training on the same day and for retention testing for each group (middle). Significant p-values for the main effects of intervention and timepoint (right). ANCOVA p-values for the training group and time of testing were < 0.001 and 0.013, respectively.*

The effect size of the intermittent visual occlusion training far exceeds most other dynamic balance training approaches (Lesinski et al., 2015). Lesinski et al. (2015) provided an extensive review of effect sizes of balance training in young health subjects and found that between subjects’ effect sizes of 5 dynamic balance studies showed medium to large effects (mean effect size = 0.92). Only one study showed very large between subject’s effect sizes (=3.68) that exceeded these of our study (Rasool and George, 2007). However, training was performed over a course of 4 weeks with 5 training sessions per week and a protocol that progressed from simple static to more complex dynamic balance tasks. Our study included only one 30-min training session and thus cannot be directly compared with improvements resulting from multiple training sessions over a longer period of time.

Our results showed a much larger improvement in motor performance outcomes compared to previous studies using electrically controlled liquid crystal glasses for sport skill training. These sports studies (Holliday, 2013; Mitroff et al., 2013; Hülsdünker et al., 2019) used a relatively high frequency visual perturbation, with an occlusion frequency at approximately 10 Hz. Our perturbation was roughly 0.1 Hz. The previous studies with high frequency perturbations support a link between visual stimuli and motor learning (Wilkins and Appelbaum, 2020), but our study with low frequency perturbations had a much larger training effect. Using repeated low frequency visual perturbations might result in cross-modal synchronization leading to more effective multisensory processing and integration (Lakatos et al., 2008; Schroeder and Lakatos, 2009; Thorne et al., 2011). Cross-modal synchronization refers to when perturbation in one sensory channel leads to synchronization of neural oscillations across different sensory modalities. The low frequency visual perturbation may lead to synchronization in visual, vestibular, and proprioceptive neural oscillations. As a result, the acuity of sensory integration might be enhanced (Lakatos et al., 2007; Thorne et al., 2011). Future studies should examine a range of different frequencies for visual stimuli and record neural oscillations with EEG to determine the optimal range and to examine potential neural mechanisms. The training task would have to be the same to determine which frequency is optimal and to control for changing neural control aspects.

There were several limitations to this study. We only examined young, healthy subjects, and did not test older subjects or subjects with neurological impairments affecting dynamic balance. A natural extension of the work here would be to test elderly or patient populations that have a higher propensity for falling. We also did not test for transfer to other balance tasks. Balance training is known for being very task specific (Kümmel et al., 2016). There is no information from this study whether the training methodology of intermittent visual occlusions would lead to improvements in balance for tasks like overground walking or running, or for standing posture. We did not examine long-term training effects, nor did we determine if multiple sessions of training with intermittent visual occlusions leads to additive improvements in balance performance. Despite these limitations, the results provide strong support for the beneficial training effects of using intermittent visual occlusions for task-specific dynamic balance training.

With falls affecting the health and quality of life of millions of people around the world, intermittent visual occlusions are a relatively simple approach for enhancing balance training interventions. The cost, storage, and ease of use of the glasses makes them scalable to a large number of individuals. Different types of exercise are used as balance training interventions to reduce falls amongst risk populations, such as Tai Chi, Yoga, and Pilates (Wolf et al., 1996; Gatts and Woollacott, 2007; Jeter et al., 2014). Future studies should look at the possibility of adding intermittent visual occlusions to such exercise classes, in community centers, and even retirement homes to assess balance improvements in larger populations.

## Data Availability Statement

The original contributions presented in the study are included in the article/supplementary material, further inquiries can be directed to the corresponding author.

## Ethics Statement

The studies involving human participants were reviewed and approved by IRB University of Florida. The patients/participants provided their written informed consent to participate in this study.

## Author Contributions

E-RS and DF co-designed this study. E-RS acquired and analyzed the data and drafted the manuscript. DF contributed to data interpretation and manuscript drafting. Both authors have read and approved the final manuscript. No one who qualifies for authorship has been omitted from the list.

## Conflict of Interest

The authors have a patent pending related to the training method.

## Publisher’s Note

All claims expressed in this article are solely those of the authors and do not necessarily represent those of their affiliated organizations, or those of the publisher, the editors and the reviewers. Any product that may be evaluated in this article, or claim that may be made by its manufacturer, is not guaranteed or endorsed by the publisher.
